# Supplementary material for: Complexome profiling of the Chlamydomonas psb28 mutant reveals TEF5 as an early PSII assembly factor
Source: Plant Cell. 2025 Mar 17;37(6):koaf055. doi: 10.1093/plcell/koaf055 (PMC12164590; doi:10.1093/plcell/koaf055)
Supplement: koaf055_Supplementary_Data [file koaf055_supplementary_data.zip › psb28_interactive.zip!/plots/intensity/qualityPlots/peptides/TATB_Cre08.g371650.t1.2.html]

### Cre08.g371650.t1.2

Trivial name: TATB  
SeqCoverage [median]: 24.50  
Mass: 25.49 kDa  
  
 

| Sequence | MissedCleavage | Mass [Da] | wtIntensity | muIntensity | Score |
| --- | --- | --- | --- | --- | --- |
| EAARPAPRPVSEATGVSDTMAK | 2 | 2240.12 | 2622.37 | 0.00 | 43.61 |
| EVVQVSQELK | 0 | 1157.63 | 2108.91 | 7104.63 | 166.09 |
| GTLESELGINELR | 0 | 1429.74 | 2045.17 | 1936.58 | 118.23 |
| PAPRPVSEATGVSDTMAK | 1 | 1812.90 | 0.00 | 426.70 | 43.21 |
| PVSEATGVSDTMAK | 0 | 1391.66 | 47683.27 | 150719.84 | 110.08 |
| TFQPTIK | 0 | 833.46 | 1588.66 | 838.71 | 125.25 |
| VLDPEIEAK | 0 | 1012.54 | 3639.33 | 2258.84 | 74.13 |
| VLDPEIEAKR | 1 | 1168.65 | 3247.38 | 7529.72 | 81.34 |
|  |  |  | 62935.1 | 170815.0 |  |
